# The experience of “at‐risk” status for familial frontotemporal dementia (fFTD) and its impact on reproductive decision‐making: A qualitative study

**DOI:** 10.1002/jgc4.2000

**Published:** 2025-01-12

**Authors:** Neil Fahy, Oliver S. Hayes, Caroline V. Greaves, Sophie E. Goldsmith, Emilie V. Brotherhood, Jonathan D. Rohrer, Emma Harding, Joshua Stott

**Affiliations:** ^1^ Camden Learning Disability Service, London Borough of Camden London UK; ^2^ Research Department of Clinical, Educational and Health Psychology University College London London UK; ^3^ Doctor of Clinical Psychology Programme University of Liverpool Liverpool UK; ^4^ Dementia Research Centre, UCL Queen Square Institute of Neurology University College London London UK; ^5^ Adapt Lab University College London London UK

**Keywords:** decision‐making, dementia, frontotemporal dementia, genetic disorders, lived experience, thematic analysis

## Abstract

Familial frontotemporal dementia (fFTD) is an autosomal dominant heritable form of FTD, onsetting in mid‐life, characterized by behavioral and personality changes. Children of an affected parent are at 50% risk of inheriting the relevant fFTD gene variant and developing FTD. Genetic testing means a growing group of people are aware of or considering learning their risk status. This knowledge, combined with witnessing parents' symptoms, has implications for reproduction. This study explores attitudes and approaches to reproductive decision‐making among those at risk for fFTD. Thirteen qualitative interviews were conducted with at‐risk individuals, including parents and non‐parents, and analyzed using Thematic Analysis to explore experiences of at‐risk relatives of people with symptomatic FTD, attitudes toward reproductive decision‐making, and, among parents, influences of genetic risk status on parenting. The themes identified were: (1) Fear of repetition of own experience with symptomatic relatives; (2) Approaches to mitigating repetition; (3) Responses to genetic risk in reproductive decision‐making; (4) Accounting for timing in reproductive decision‐making; (5) Challenges of disclosing genetic risk to children; (6) Other mitigating factors in reproductive decision‐making. Findings highlight the key role of previous experiences with symptomatic relatives in shaping attitudes toward genetic risk status and approaches to managing it in reproductive decision‐making. Findings highlight a need for responsive genetic counseling focused on exploring options alongside providing information and signposting to practical legal and financial support. Future research should specifically compare experiences in fFTD with experiences in other heritable neurodegenerative disorders and explore reproductive decision‐making for couples where one partner is at risk of fFTD.

**What is known about this topic**

We know very little about reproductive decision‐making in individuals with familial frontotemporal dementia, with much inferred from similar conditions such as Huntington's disease. What we do know is that uptake of genetic testing is low (Greaves & Rohrer, 2019) and that family members are often responsible for the care of affected persons.

**What this paper adds to the topic**

This paper adds to the knowledge base by marking the first formal investigation into the views and attitudes toward reproductive decision‐making among those at risk for fFTD, focusing on the connections drawn between previous experiences with affected relatives, overall views on risk, and current views and intentions regarding reproduction.

## 1 INTRODUCTION

Frontotemporal dementia (FTD), sometimes called Pick's disease, is a neurodegenerative disease affecting the frontal and temporal lobes (Sivasathiaseelan et al., 2019). It manifests clinically in several variants: a behavioral variant (bvFTD), characterized by impaired emotional regulation, executive functioning, inhibition, and empathy; and a number of language variants, known as the primary progressive aphasias (PPA) including semantic variant PPA, characterized by impaired semantic memory and non‐fluent variant PPA, characterized by difficulties with language production (Warren et al., 2013). Age of onset is on average between 50 and 60, but can occur earlier in the 30s and 40s, as well as later in life, even into the 90s (Warren et al., 2013). In around a third of all cases, and more than 40% of behavioral variant cases, an autosomal dominant inheritance pattern emerges (Rohrer et al., 2009), whereby any child of a parent with implicated genes (most often *C9orf72, GRN, MAPT*) has 50% chance of developing FTD; these cases can be classified as “familial Frontotemporal dementia (fFTD).” There are several genes associated with fFTD: more than 40 mutations in the Microtubule associated protein tau (*MAPT*) gene, linked with chromosome 17 and the build‐up of tau proteins in glial cells and neurons (Seelaar et al., 2011) are seen in up to 20% of fFTD cases (Olszweska et al., 2016); approximately 60 mutations in the progranulin gene (*GRN*) are observed in between 5% and 22% of fFTD cases (Olszweska et al., 2016; Seelaar et al., 2011), and affects the encoding of the homonymous protein; differences in the *C9orf72* gene, which lead to extended repeat intronic expansion are observed in a further 25% of fFTD cases (Olszweska et al., 2016). Outside of these primary implicated genes, there is a range of less common variations associated with less than 5% of fFTD cases (e.g., *VPC, CHMP2B, TARDP, FUS, ITM2B, TBK1, TBP*) (Greaves & Rohrer, 2019). Due to the above‐outlined variability in age‐related penetrance in fFTD, and particularly due to its high intrafamilial variability (Greaves & Rohrer, 2019), individuals who have inherited implicated genes, and at‐risk individuals who have chosen not to pursue genetic testing, are left with significant uncertainty about the potential age of onset and trajectory of symptomology. Similarly, motor neurone type symptomology is also variably associated with several fFTD‐implicated genes, particularly *C9orf72*, and can lead to an overlapping syndrome of FTD‐related amyotrophic lateral sclerosis (FTD‐ALS) (Greaves & Rohrer, 2019; Turner et al., 2017). The possibility of inheritance of FTD‐ALS symptomology has the potential to further complicate the decision‐making of at‐risk individuals.

Predictive testing is available to first‐degree relatives of affected individuals, though uptake is generally low (Greaves & Rohrer, 2019). No treatments are available (Manoochehri & Huey, 2012; Rosness et al., 2016; Sivasathiaseelan et al., 2019; Warren et al., 2013), and management focuses on behavioral symptom control and practical support for affected individuals and carers (Warren et al., 2013).

Daily symptom management is largely the responsibility of family carers. Given the variability in symptom progression, and interpersonal challenges of characteristic symptoms of bvFTD (Caceres et al., 2016; Massimo et al., 2013), caring causes greater carer stress than in Alzheimer's Disease (Kaiser & Panegyres, 2006; Riedijk et al., 2006), and is associated with increased rate of mental and physical health issues (Caceres et al., 2016; Wong & Wallhagen, 2014). Accessing appropriate support is frustrating (Bruinsma et al., 2022), and caring in FTD is therefore complex and demanding (Tookey et al., 2022; Van Vliet et al., 2011).

One reason some at‐risk individuals choose to undergo genetic testing is to inform their reproductive decision‐making. This consideration is highlighted both in the context of fFTD and in other conditions (e.g., Crook et al., 2021).

At‐risk individuals (who don't know their genetic status or who are gene‐positive) will have different preferred options to mitigate passing their risk to their biological children depending on their values and priorities. Conception without intervention can lead to worries about the current and future impact of the disease on the self, children, and relationships, as well as the risk of transmission to biological children. Pre‐natal diagnosis (PND)—genetic testing of an established pregnancy—with an option to terminate affected pregnancies, can allow for conception without intervention and can be quicker, but can be emotionally challenging due to potential termination. Pre‐implantation genetic testing (PGT)/pre‐implantation genetic diagnosis (PGD)—implantation of non‐carrier IVF embryos—avoids termination, but is financially, physically, and emotionally demanding with a relatively low success rate (de Die‐Smulders et al., 2013). Non‐biological parenting approaches (via adoption, fostering, or donors) are often considered but rarely utilized (de Die‐Smulders et al., 2013), perhaps owing to concerns about having a non‐biological relationship to the child, coupled with uncertainty relating to the physical and mental health of adopted children (Severijns et al., 2021). Alternatively, abstaining from having children, which avoids transmission to biological children, is associated with ongoing rumination and dissatisfaction where a strong desire for children is present (Frets et al., 1990).

In the absence of fFTD‐specific research, studies investigating reproductive decision‐making in the context of genetic risk of other heritable conditions highlight the complexity of the topic. Transmission to biological children is a common concern for those at genetic risk for many heritable conditions (Hershberger et al., 2012; Severijns et al., 2021). For example, Huntington's disease (HD) is the most directly comparable genetic condition to fFTD. It is a neurodegenerative disease demonstrating an autosomal dominant inheritance pattern and characterized by psychiatric symptoms, personality changes, and motor issues, with an average age of onset of between 30 and 50 (Roos, 2010). Desire to have children for those with HD is relatively common, but potential disease transmission to biological children is often reported as a major influence on reproductive decision‐making and views of assistive options, especially PGT are generally positive, but uptake of testing and risk mitigation options remain relatively low (Fahy et al., 2023). The research identified in the above review indicates that the process of balancing perceived responsibility to prevent HD transmission with a longstanding desire for children is challenging, with some at‐risk individuals responding by accepting genetic risk as one among many their children will face, and others maintaining optimism for future treatments. Rumination and uncertainty over reproductive decisions were commonly reported, often influenced by implicit or explicit judgment of reproductive choices by family, healthcare professionals, and wider social narratives.

Genetic testing for HD is well‐established and represents a gold standard approach to testing and management of associated symptoms (MacLeod et al., 2013). The protocol has been applied to fFTD due to relative similarity (Greaves & Rohrer, 2019). Independent research to explore the specific experience of those at risk of fFTD will be beneficial in identifying any adaptions needed to the HD protocol.

In summary, fFTD is an autosomal dominant, progressively deteriorating condition (Greaves & Rohrer, 2019). Its characteristic symptoms profoundly impact quality of life for the affected individuals and their network, and the impairment of complex behavior and age of onset mean parenting ability could be affected (Tookey et al., 2022; Van Vliet et al., 2011). Those at genetic risk therefore often have significant experience with an affected parent and are likely to be acutely aware of their own potential risk. It seems reasonable to assume that these factors might meaningfully impact at‐risk individuals' views of their own risk, and also their attitudes toward reproductive decision‐making, both in terms of potential transmission to biological children as well as potential caring burden as in HD (Fahy et al., 2023). However, there is a lack of research regarding these factors in fFTD.

We aimed to explore views and attitudes toward reproductive decision‐making among those at risk for fFTD focusing on the connections drawn between previous experiences with affected relatives, overall views on risk, and current views and intentions regarding reproduction. We also explored if risk status has influenced the approach to parenting among those with children, and the future reproductive intentions of those without children. Given the exploratory nature of this study and the subjective, experiential nature of the topics of concern, a qualitative approach was used, so participant experiences and views formed the basis of developed themes.

## 2 METHOD

### 2.1 Participants and setting

We recruited individuals at‐risk (currently asymptomatic) for fFTD. This was done by convenience sampling of the ongoing cohort Study A, whose ethics this study operated under. In terms of inclusion criteria, we included all people within the at‐risk group who were currently symptomatic, with at‐risk being defined as having a first‐degree relative (parent or sibling) with a confirmed fFTD diagnosis (Greaves & Rohrer, 2019). Lack of fluency in English or experiencing current FTD symptoms preventing the provision of informed consent were exclusion criteria.

### 2.2 Recruitment and data collection

Participants were recruited from the cohort of the University A arm of Study A, an ongoing cohort study monitoring outcomes for those at risk of fFTD. Sampling was purposive to achieve a balance of parents and non‐parents, and a range of genetic risk knowledge statuses. Participants were recruited between 28/04/2021 and 24/01/2022. All participants provided written informed consent prior to participation in the study in line with Study A ethics protocols.

Potential participants meeting inclusion criteria were identified and contacted by the University A Study A research team with information about the focus of research and an outline of topics to be covered in the interview. Interested participants then contacted University A Study A staff to arrange a convenient time for interview, which was then confirmed with the interviewer. Participant interviews were conducted remotely using Zoom or MS Teams software according to participant preference. Demographic information was collected and is presented in Table 2 in the results section.

### 2.3 Semi‐structured interview

Semi‐structured interviews covered participant's views on reproductive decision‐making and parenting in the context of genetic risk, and their experiences with fFTD‐affected relatives, as well as how these might influence their views on reproduction and parenting with the opportunity to share any additional information they thought was relevant (see Appendix S1 for full interview schedule). The interview schedule was developed through a review of relevant literature related to fFTD and other comparable neurodegenerative diseases, especially HD (Fahy et al., 2023). Input on the draft schedule was provided by members of the Study A research team and three experts by experience and was updated in line with this feedback for clarity, sensitivity, and neutrality of wording.

Interviews were recorded and transcribed verbatim, with identifying information (e.g., names, place names) removed. Interview length ranged from 40 to 75 min.

### 2.4 Analysis

Transcripts were analyzed using Braun and Clarke's Thematic Analysis (2006, 2013). This qualitative analysis approach involves the exploration of data at the level of units of semantic meaning (“codes”), developed or organized into themes based on patterns of similarity and difference. The six analysis stages (Braun & Clark, 2006), undertaken using NVivo for Windows PC, are outlined in Table 1.

**TABLE 1:** Six stages of thematic analysis adapted from Braun and Clarke (2006, 2013).

Stage	Description
1	Immersion and familiarization with data set, via transcription, repeated reading and memo‐ing
2	Initial coding, where each relevant portion of data is assigned a code
3	Development of initial themes via comparison and grouping of codes according to relevance and significance to research aims
4	Review of initial themes against included codes and wider data set
5	Definition of reviewed themes, to clearly outline included concepts and relationships between them
6	Reporting of results, illustrating themes and sub‐themes with relevant quotations from transcripts, and offering summative responses to initial research questions

The epistemological stance of descriptive phenomenology was taken. This focuses on understanding and reporting individual experiences as reported and understood by participants, compared to interpretative phenomenology, which offers interpretation of this experience by the author (Sundler et al., 2019). The descriptive stance seemed appropriate to this study's aims of eliciting novel information about at‐risk individuals' experience and meaning‐making to form a basis for greater understanding of reproductive decision‐making. Given the relatively novel nature of this research, the study aimed to utilize a hybrid approach, whereby deductive generation of broad code categories informed by previous research in HD was complimented with novel codes identified in the process of analysis. This approach has been effectively employed in qualitative research regarding reproductive decision‐making in other genetically heritable conditions (Gong et al., 2016; Kazmerski et al., 2017; Quaid et al., 2010).

### 2.5 Ethics

This project was conducted with appropriate ethical approval. All transcripts were pseudonymized for the purpose of participant confidentiality. Any expression of topics relating to suicidal ideation was handled in accordance with Study A protocols, which are subject to ethical review. When expressed, if participants were not currently under the care of a mental health professional, they were signposted to relevant resources and if needed referred to a mental health professional for review. Involved researchers underwent supervision in which they could discuss the event.

## 3 RESULTS

### 3.1 Demographic features

The sample of 13 participants was majority female (*n* = 8), with an age range of 27–61; all participants were White British and none of the participants were related. At the time of interview, six participants were parents and seven were childless. Six participants had received a positive genetic test, five had chosen not to pursue a test, one had agreed to testing and was awaiting an appointment, and one had a negative genetic test. Mean time since testing for those pursuing was 5 years (Range: 1–15, SD = 4.8) (see Table 2 for a summary of participant characteristics).

**TABLE 2:** Participant demographics.

Code	Age	Sex	Ethnicity	Genetic test status	No. of children	Children before or after risk knowledge?	No. of affected relatives	Years in education
P1	41	M	White British	Positive	0	N/A	1	15
P2	34	M	White British	Not yet tested	0	N/A	2	16
P3	38	M	White British	Not yet tested	2	After	1	16
P4	35	F	White British	Positive	2	One before, one after	1	16
P5	38	F	White British	Positive	0	N/A	3	17
P6	61	F	White British	Not yet tested	1	Before	2	14
P7	28	F	White British	Not yet tested	0	N/A	1	16
P8	45	F	White British	Positive	3	Before	1	17
P9	58	F	White British	Positive	3	Before	3	12
P10	39	M	White British	Positive	0	N/A	1	13
P11	39	F	White British	Negative	0	N/A	1	16
P12	39	F	White British	Not yet tested	0	N/A	1	17
P13	42	M	White British	Not yet tested	3	After	1	14

### 3.2 Themes

Six core themes were identified: (1) Fear of repetition of own experience with symptomatic relatives; (2) Approaches to mitigating repetition; (3) Responses to genetic risk in reproductive decision‐making; (4) Accounting for timing in at‐risk reproductive decision‐making; (5) The challenges of disclosing genetic risk to children; and (6) Other mitigating factors in reproductive decision‐making (see Table 3 for the breakdown of themes across participants).

**TABLE 3:** Representation of participants by theme.

Themes		P1	P2	P3	P4	P5	P6	P7	P8	P9	P10	P11	P12	P13
**1**	**Fear of repetition of own experience with symptomatic relatives**	x	x	x	x	x	x	x	x	x	x	x	x	x
*1.1*	*Process of becoming symptomatic*	x	x	x	x	x	x	x	x		x	x		x
*1.2*	*Caring burden*	x	x	x	x	x		x	x	x	x	x	x	x
**2**	**Approaches to avoiding repetition**	x		x	x	x	x	x	x	x	x	x	x	x
*2.1*	*Practical planning to mitigate worst aspects*	x		x	x	x	x							x
*2.2*	*Emphasis on parenting “in the here and now” (Parents only)*			x	x		x		x	x				x
*2.3*	*Research Involvement*	x		x	x	x	x	x			x	x	x	x
*2.4*	*Suicide as a potential response to future FTD emergence*				x		x				x			
**3**	**Responses to risk in reproductive decision‐making**	x	x	x	x	x	x	x	x	x	x	x	x	x
*3.1*	*“I want to be part of the reason the gene disappears”: Risk minimization*	x	x			x		x			x	x	x	
*3.2*	*“It sucks, but I can't rule everything out”: Risk acceptance*			x	x		x		x	x				x
**4**	**Accounting for timing in at‐risk reproductive decision‐making**	x				x		x			x	x	x	x
**5**	**The challenges of disclosing genetic risk to children**	x	x	x	x		x	x	x	x	x	x		x
**6**	**Other mitigating factors in reproductive decision‐making**	x	x			x	x	x			x	x	x	

*Note*: Main themes in bold, subthemes in italics.

#### 3.2.1 Theme 1: Fear of repetition of own experience with symptomatic relatives

Here participants connect experiences with their symptomatic relatives with concerns about their potential replication for themselves and their loved ones. Most participants, and all who had significant contact with an affected relative, described practical challenges and emotional strain.

##### Process of becoming symptomatic

Many participants described the challenges of disease progression for affected relatives—from relatively benign behavioral eccentricities to interpersonally challenging loss of inhibition and empathy, to dangerous unpredictability and vulnerability, to emotionally distressing loss of communication ability and independence.

Many participants highlighted a sense of loss of the affected relative due to such changes in behavior and personality, for example with a loss of meaningful communication:

P2 (34, M, Not yet tested) It was basically like very little human left of him because he… just didn't seem to understand anything that was going on.

Several participants highlighted fears they would experience a similar “loss of self” in their own future due to their genetic status:

P11 (39, F, Negative) The thing that was upsetting me the most when I… got my result—that I could behave in a completely different way and lose my identity.

Some participants emphasized the potential impact on partners and children of witnessing their potential disease progression as a significant fear in and of itself, irrespective of whether the gene was passed on:

P1 (41, M, Positive) Not only would I be passing it on, I'd be dying early and they'd have to see that, it'd be terrible. So even if I didn't pass it on, it'd be awful.

##### Caring burden

Many participants discussed the practical and emotional caring burden of supporting someone with fFTD, and the anticipation of their own children or families having to also face this was additionally worrying. Particularly difficult aspects included navigating complex diagnostic, care, and legal issues, managing challenging behaviors, loss of relationship with affected person, and a loss of own plans and priorities as a result of caring, associated with frustration, resentment, and resultant guilt. P4 highlights these challenges, and the reversal of the typical parent–child relationship:

P4 (35, F, Positive) Basically he soiled […] himself and was wandering around the house […]. I had to shower him, which I had never done… I thought, “oh my God, I'm showering my dad”.

Some participants suggested the caring role may be more challenging than experiencing symptoms, due to a common lack of insight, for example:

P4 (35, F, Positive) I think that with FTD, the suffering is more on the carer than the person. Because… you lose the emotions …So not in the hard part having being symptomatic, so everyone around you has to manage that.

Several participants explicitly highlighted the desire to avoid such caring burden for their families generally, and children specifically:

P5 (38, F, Positive) But I wouldn't really want them to have to experience what I experienced …

#### 3.2.2 Theme 2: Approaches to mitigating repetition

Having witnessed the impact of fFTD witnessed for their affected relatives in their own lives, participants adopted various approaches to avoiding or mitigating this impact for themselves and their children.

##### Practical planning to mitigate worst aspects

Many participants discussed the specific challenges caused by fFTD's unexpected emergence and unpredictable trajectory—lasting power of attorney was rarely in place, complicating care, and financial decision‐making. Where pre‐arranged it offered some relief, and where not resultant protracted legal issues were a significant source of stress, which impacted participants' own future planning:

P5 (38, F, Positive) Soon as I have anything to leave, the will is getting done…if there's one moral of my mother's story, I think it's that.

Some participants made decisions explicitly to protect their families financially in case of their illness via comprehensive life insurance, legal safeguards against care costs, or prioritizing career decisions that maximized current earning power against future need:

P3 (38, M, Not yet tested) I have critical illness life cover, I have a good job with a good wage, they [children] have a good life, and so if I'm not able to carry on with that I wouldn't want them to be… affected.

##### Emphasis on parenting “in the here and now”

Many current or prospective parent participants reported the impact of their experience with affected relatives on parenting approach, with several prioritizing maximizing time spent with children and “making memories”:

P4 (35, F, Positive) I definitely have a different approach of live now, like if I can spend longer at home with my kids, so I work flexibly I've definitely put more of an emphasis…and a focus on that. And my husband has done the same. Our approach is… Don't lose out on the now stuff.

Some reported efforts to communicate “life lessons” to children, and share advice that they may not be present to share in the future:

P13 (42, M, Not yet tested) But I feel as though…when I'm with our children…I'm trying to convey life lessons onto them…feel a wee bit more thorough as a parent.

##### Research involvement

All participants emphasized the importance of fFTD‐related research involvement. Many viewed it as a positive, productive response to living at risk, reducing its negative impact. Several viewed research participation as potentially facilitating treatment development, and therefore reducing the impact of fFTD on future lives:

P6 (61, F, Not yet tested) I've opted to be part of the Study A research. Because…The sooner we can find treatments for this gene mutation, then…FTD would be something that could be treated.

Several parent participants explicitly viewed research participation as something undertaken for their children's benefit, hoping that potential treatments will minimize the role of fFTD in their children's lives:

P3 (38, M, Not yet tested) …it could benefit me, and I think it will absolutely benefit [my children] if it needs to…

##### Suicide as a potential response to future FTD emergence

A subset of participants explicitly considered plans to end their lives in the future should they develop fFTD. Some intended to pursue plans reflexively to symptoms emerging, and others aimed for specific age milestones, often estimated from affected relatives' disease trajectories. All participants in this subset had discussed their plans with close relatives, either partners or adult children, who were understanding and supportive. Some wished to avoid repeating their affected relative's experience:

P10 (39, M, Positive) Because I don't really want to get ill…I personally wouldn't see the point in being around if I'm honest.

For other participants, the main motivator was to avoid placing a caring burden on their children in the future, should they become symptomatic:

P4 (35, F, Positive) And I would do anything I could to alleviate that [caring burden] for them. I sort of have a bit of a backup plan in my mind that I would aim to do something along the lines of Dignitas or some sort of end before the end. And then they don't have to take that.

All participants discussing this topic emphasized these as potential options being considered in the far future related to future fFTD symptomology, denying suicidal ideation, intent, or plan in the present or near future. Participants characterized this option as reassuring to consider as an “*escape hatch (P4)*” in their personal worst‐case scenario.

#### 3.2.3 Theme 3: Responses to risk in reproductive decision‐making

All participants viewed risk knowledge as significant and at times challenging, with most describing its inevitable impact on life‐planning:

P7 (28, F, Not yet tested) Obviously, the whole FTD thing comes into it because it's – you either make a kind of conscious choice to address it, or it's a conscious choice to not.

Many participants emphasized the highly personal nature of reproductive decisions. Outside judgment of their or others' chosen options was experienced as unwelcome and unhelpful.

##### “I want to be part of the reason the gene disappears”: Risk minimization

Many participants categorically rejected reproductive outcomes that might allow for further fFTD transmission to biological children, often expressing their long‐standing ambivalence toward having children before becoming aware of being personally at risk for fFTD. Motivators for this view included desire to end fFTD transmission to biological children or avoid caring burden, and anticipated guilt were a future child to struggle with genetic risk themselves:

P1 (41, M, Positive) I don't want to spread the gene at all…

There was a range of expressed intensity in attitudes to risk avoidance. Some viewed PGT/PGD as an acceptably risk‐free approach:

P10 (39, M, Positive) If I was going to have children, that's the woman I would like to have children with, but as well because you had the PGD, there was an option there… [we] grabbed it with both hands.

Some considered adoption or fostering as a way to avoid transmission to children; however, most ultimately decided against due to ongoing concerns about caring burden. Some participants took a more absolute stance, whereby they took all steps deemed necessary to avoid transmission of the disease to biological children and caring burden, up to and including avoiding romantic relationships:

P2 (34, M, Not yet tested) Well [pause] I'm in a situation now where I have zero dependents, and… I guess if I was already in a relationship with someone who wanted kids, then you know we'd have a conversation but… since I'm in a position where I've got no dependents, if I'm at‐risk why would I want to introduce those?

##### “It sucks, but I can't rule everything out”: Risk acceptance

Conversely, many participants viewed fFTD as a serious issue, but one among many potential risks children may face, and not in itself sufficient to avoid having children:

P4 (35, F, Positive) The other thing that was quite big for both of us, … yes, I could get it. Yes, my child could get it, but we could also get a million other things. We could also get hit by a bus. We could also get cancer. We could also have heart disease. So we kind of went with. Yeah, it sucks, but I can't rule everything out.

This view was common among those with children at interview, or with a long‐standing desire to have children. Some stressed the importance of avoiding risk knowledge controlling their lives or preventing important life goal attainment:

P3 (38, M, Not yet tested) You wouldn't want to do anything if you let it consume you in that way and kind of let it prevent you from doing. What's the point in living then, anyway, if you're not going to do the things that you want to do?

For those who had children before learning about their fFTD risk, there was an expressed need to “make peace” with previous reproductive decisions made in the context of imperfect knowledge:

P9 (58, F, Positive) I was very philosophical about it because I said to deny the fact that I might get Huntington's disease, not knowing about the Pick's bit, is to deny my family. And how can you deny your family? That's my genes… That's me.

Other participants found this more challenging. One participant described the process as immediate acceptance upon getting their test result, while another described a gradual, partial process where acceptance existed alongside regret at the lack of knowledge at the time. A third described the process as an intensely painful and irresolvable alternation between partial acceptance and ruminative guilt. One participant added more hopefully the possibility that their children would have greater treatment options than they did:

P5 (38, F, Positive) I think it's extremely likely if I had a child today, there would be a treatment for them in 50 years' time. Especially now, I do think that's much more likely to be the case.

#### 3.2.4 Theme 4: Accounting for timing in at‐risk reproductive decision‐making

Many participants, regardless of parental status or decisions regarding genetic testing, identified that fFTD risk introduced a perceived sense of time pressure on reproductive decision‐making. Many described using the age of onset of their affected relative as an estimated “time limit” regarding their ability to effectively engage in parental responsibility before it might be impaired. For some, this awareness was a dis‐incentivizing factor. They described calculating the age at which they would need to have children for them to reach adulthood by potential age of onset. Reaching this age without children, they then accepted that biological parenthood would not occur:

P10 (39, M, Positive) Now, like I say, I'm 39 now. So I'm at that age now where …even if we were successful on the third cycle for myself I think that's the limit really and we've got closure on it now.

For others, this awareness acted as a motivating factor, with similar age calculations leading to proactive pursuit of relationships, marriage, and parenthood within this time frame, to maximize their available time for active parenting and involvement in their children's lives:

P3 (38, M, Not yet tested) So my father was 40 when I was born, and I'm the eldest. He didn't meet any of his grandchildren because he started having kids a lot later. I didn't want to do that.

Some participants described this awareness of time limits leading to solidification of a long‐standing vague disinterest in parenthood. For these participants, this time limit instigated a more active process of consideration than might otherwise have been the case, but ultimately led to a re‐affirmation of already‐held views:

P5 (38, F, Positive) Obviously … It [genetic risk] has an importance in so far as emphasis of the timing, when I made some of the decisions made around it. But I do not think at all that it has any definitive part to play in any of the decisions I've made around it.

#### 3.2.5 Theme 5: The challenges of disclosing genetic risk to children

The majority of participants, both with and without children agreed that the idea of discussing fFTD risk transmission with their children was intensely challenging. For some participants without children, the challenge of this influenced the decision not to have children. Common concerns regarding disclosing risk to children included uncertainty in the child's ability to cope with challenging knowledge, potential damage to the quality and strength of the parent–child relationship, fundamental disagreement by the child with the parent's choices regarding reproduction, and a resulting uncertainty in how to broach the issue for example:

P8 (45, F, Positive) now my daughter's 18 and I should really tell her what I found out. And I don't know how the hell to do that. I just can't face it.

Regarding disclosure, some participants described feeling trapped between a sense of knowledge being important for their child's life planning, and the distress of their anticipated reactions. This could lead to disclosures in unplanned situations which could lead to distress for both parties, e.g.:

P9 (58, F, Positive) in the end…he met a girl and they bloody got pregnant …So then he come round to tell us we're expecting and I basically told him, well, I've got this and you might have to terminate your baby and, test your baby, and…he basically lost the plot and left the room.

Several participants with younger children described a sense that their children would at some point approach them with questions regarding fFTD, at which point it would be discussed. The decision to wait for children to ask was viewed in terms of enabling autonomy, and avoiding over‐burdening young children with distressing, complex information they would struggle to understand. Some participants had a general idea of telling their children if not asked by a certain age:

P3 (38, M, Not yet tested) I think at the moment, they don't understand you know, they ask where my father is but they wouldn't understand. I think their teens might be a time, but if one of them's 10 years old and specifically asks you the question then you'd probably answer it.

#### 3.2.6 Theme 6: Other mitigating factors in reproductive decision‐making

Several participants, both parents and non‐parents, emphasized that fFTD risk was not the only and/or single deciding factor influencing reproductive decision‐making. Among those without children or undecided, it was common for desire to have children to be conditional on factors such as a security of relationship, housing and income, partner's views, and a desire to maintain current lifestyle. These factors often played an equal or greater role in fFTD risk in reproductive decision‐making. For example:

P10 (39, M, Positive) so I'm, I'm happy in my relationship with my wife…for me [having a child] is something that she wanted…and so did I…But if we couldn't, in my view…it'd be okay either way.

Conversely, among those with children, some identified factors played a significant role in reproductive decision‐making alongside fFTD risk, including perceived suitability as a parent, strong pre‐existing desire to have children, and a personal valuing of family life. Some participants, even without children, stressed that they had made their reproductive decisions before risk knowledge was in the picture, and did not intend to change them:

P4 (35, F, Positive) while I was going through that [genetic testing] process, we decided this is the right time for us [to have a child] and that isn't going to be a contributing factor. …So we did.

## 4 DISCUSSION

This study was the first to our knowledge to explore the relationship between fFTD risk and reproductive decision‐making and parenting. We identified six interacting themes: Fear of repetition of own experience with symptomatic relatives; Approaches to mitigating repetition; Responses to genetic risk in reproductive decision‐making; Accounting for timing in at‐risk reproductive decision‐making; The challenges of disclosing genetic risk to children; and Other mitigating factors in reproductive decision‐making. Overall themes suggested a complex relationship to fFTD risk, and a multi‐faceted and varied approach to it in reproductive decision‐making, which incorporated but was not determined by fFTD risk. These themes connect with and, often, extend existing literature in other conditions.

In particular, qualitative research on reproductive decision‐making generally (Beeson & Golbus, 1985; Hershberger et al., 2012; Severijns et al., 2021), in HD (Klitzman et al., 2007; Quaid et al., 2010), cystic fibrosis (Kazmerski et al., 2017), ALS (Hartzfeld et al., 2015), and BRCA inheritance (Quinn et al., 2010) commonly report participant concerns regarding transmission to biological children as significantly affecting reproductive decision‐making. This study found comparable concerns; however, participants placed an additional, similarly significant emphasis on the impact of caring burden on their own lives and a desire to avoid this for children independent of transmission of the condition to them. The desire to avoid caring burden, additionally to concerns about witnessing parent symptomology and fears about transmission to biological children, is rarely expressed in research regarding other genetically heritable conditions, though it sometimes emerges in studies on HD (Klitzman et al., 2007). Given that fFTD is associated with personality change, similar to HD, caring burden in fFTD has specific interpersonal and emotional challenges not always present in other, primarily physical health conditions. It appears that these experiences have a lasting impact on participants' views on what a future living with fFTD would be like, and therefore a resultant desire to lessen impact on others in similar ways to HD.

Findings also highlighted that participants viewed fFTD risk as one factor within many influencing reproductive decision‐making, and rarely appears to alter intentions completely. However, it did appear to be influential on participants' *strategic approach* to reproductive and risk mitigation, for example through utilization of PGT and practical legal and financial planning. This modification of specific approach in light of risk knowledge is also reported in HD (Klitzman et al., 2007) and in BRCA inheritance (Quinn et al., 2010). Indeed, Severijns et al. (2021) suggest that, across several genetically heritable conditions, reproductive decision‐making is a complex process requiring consideration of multiple options, with couples previously expressing child desire generally more likely to express preference for biologically related. The findings also connect to previous work on how genetic risk is conceptualized and responded to in reproductive decision‐making. For example, Quaid et al. (2010) reported a pattern in HD risk, where some individuals prioritized ending HD transmission, and others prioritized parenthood and wished to avoid HD risk “controlling [their] lives,” which was very similar to the findings reported here, with responses varying from complete risk minimization (not reproducing at all) to reproduction without taking fFTD risk into account, with many falling somewhere in the middle. This similarity across conditions may suggest that assessing appetite for risk is a key part of the process of “coming to terms with” genetic risk knowledge. This theme has implications for conversations around genetic responsibility. These considerations can be very impactful in reproductive decision‐making in the context of genetic risk as illuminated in Downing (2005).

As in previous research in HD (Klitzman et al., 2007; Quaid et al., 2010), participants appeared to use some “simplifying heuristics” (Lippman‐Hand & Fraser, 1979) in response to genetic risk knowledge. These included “risk binarization” which describes the process whereby all potential risks are pooled and considered as a total risk against the unachievable outcome of totally avoiding risk. “Scenario‐based thinking” (Lippman‐Hand & Fraser, 1979) describes the process in which the various outcomes of all available options are considered in the short, medium, and long terms. This is often done in discussion with partners, to prepare and affirm an identified “least‐lose” reproductive also option. This process is reported in relation to reproductive decision‐making in other conditions (Kazmerski et al., 2017; Quinn et al., 2010; Severijns et al., 2021).

Perhaps a newer aspect found here that connected less with previous research in other conditions was that participants identified behaviors beyond clear reproductive decisions and risk mitigation as explicitly related to reproductive decision‐making, including practical, legal, and financial advance planning, and approaches to parenting including prioritizing time spent with children and active sharing of “life lessons”. Participants emphasized that these behaviors were a result of risk knowledge, experience with affected relatives, and personal attitudes and characteristics. Similarly, research involvement was explicitly seen as a response to risk mitigation, with the aim of potential treatments reducing impact of fFTD on children. Some participants considered plans for suicide or assisted dying in the far future as a response to deterioration, again framed in terms of risk mitigation and avoidance for their loved ones of their own experiences with affected relatives.

### 4.1 Limitations

Limitations included only indirect consideration of the role of partners via second‐hard report. As reproductive decision‐making often occurs in the context of relational discussions between partners, other research highlights the inherently interpersonal, dynamic nature of this process (Myring et al., 2011; Reumkens et al., 2019a, 2019b; Severijns et al., 2021). The exclusion of partners from this study means that information about the actual process in which reproductive decisions were made will have been missed. The majority of participants were female and all were White British. This follows broad trends in research participation and cannot be said to be representative of or sensitive to differences informed by ethnic, sexual, and broader social diversity (Henrich et al., 2010; Wendler et al., 2006). Specifically, a more diverse sample might have allowed for exploration of gender, ethnic, or sexual orientation differences in reproductive decision‐making influenced by social and cultural factors, varying norms and expectations around parenthood and the processes discussed here. Several knowledge positions in relation to genetic status were represented—known positive/negative genetic status, choice not to know, and desire to know but as yet untested. Similarly, many reproductive experiences are represented—not yet actively considering children, having children knowing risk status, having children prior to risk knowledge, abstaining following risk knowledge. Though this broad representation is advisable in exploratory research, focusing exclusively on people who had children prior to risk knowledge, or exclusively on non‐parents, may have facilitated a greater level of specificity of data and findings (Palinkas et al., 2015).

Further research addressing the above limitations would be useful and research exploring the direct comparability of these experiences in fFTD and HD might be beneficial. Genetic counseling and information provision approaches for HD often form the “gold standard” in fFTD due to its long history (Greaves & Rohrer, 2019; MacLeod et al., 2013). This study found many similarities between experiences in fFTD and HD but does not necessarily support a 1:1 comparison, and further research elucidating differences could better inform practice.

Several potential clinical adaptions may be implied including expanding access to and scope of genetic counseling beyond the typical three pre‐testing sessions. Genetic counseling provision in fFTD already falls behind that available in other heritable conditions (Greaves & Rohrer, 2019), and is of variable quality (Paneque et al., 2015). Our findings suggest access to genetic counseling at important decisional intervals (e.g., when considering reproductive options) might help weigh options in a supportive non‐judgmental environment.

At present, genetic counseling broadly focuses on the provision of accurate risk information to facilitate informed reproductive decisions. Our findings suggest information alone may not be sufficient for navigating the process. It might be beneficial to support at‐risk individuals through a process of “scenario‐based thinking” (Lippman‐Hand & Fraser, 1979) to arrive at the most personally acceptable option. The emphasis placed by many participants on the benefits of proactive legal and financial planning as a way of managing distress suggests that it might be beneficial for relevant specialist healthcare centers to provide signposting and information provision regarding practical and legal planning, as well as regarding research participation, given participants' universally positive views of this option in the current study.

### 4.2 Conclusions

fFTD has profound implications for those diagnosed in their families and children. For those at risk, witnessing disease progression in their symptomatic relative can be distressing, and the experience of caring for a relative can be significantly challenging. They must also consider the possibility of their own symptom development and questions of heritability. This study provides insights into at‐risk individuals' approaches to navigating reproductive decision‐making, how risk is conceptualized, often through the lens of prior experience with affected relatives, and how multiple approaches are taken to mitigate the role of genetic risk in their own and children's lives. It suggests clinical implications for how these individuals might be supported in navigating complex reproductive decisions. It further highlights the similarities and differences in this area between fFTD and other genetically heritable conditions.

## AUTHOR CONTRIBUTIONS

All authors meet the criteria for authorship, that is, were involved in drafting the work or reviewing it critically for important intellectual content; AND gave final approval of the version to be published; AND agreed to be accountable for all aspects of the work in ensuring that questions related to the accuracy or integrity of any part of the work are appropriately investigated and resolved. Oliver S. Hayes, Emilie V. Brotherhood, and Emma Harding made substantial contributions to analysis and interpretation; Neil Fahy, Jonathan D. Rohrer, and Joshua Stott led on and made substantial contributions across the conception and design of the work; the acquisition, analysis, and interpretation of data for the work and Caroline V. Greaves and Sophie E. Goldsmith made substantial contributions to acquisition and analysis.

## FUNDING INFORMATION

The funders had no role in the design of the study; in the collection, analyses, or interpretation of data; in the writing of the manuscript; or in the decision to publish the results.

## CONFLICT OF INTEREST STATEMENT

The authors declare no conflict of interest.

## ETHICS STATEMENT

Human Studies and Informed Consent: Data was collected as part of the GENFI study for which ethical approval was obtained from the National Health Service, United Kingdom Ethics Committee, and has been awarded from University College London Hospital (Reference Number: GENFI 14/0377). The study is conducted in accordance with the Declaration of Helsinki. All participants provided written informed consent prior to enrolment in the study.

## Supporting information


Appendix S1

## Data Availability

Research data are not shared.
