# Supplementary material for: The experience of “at‐risk” status for familial frontotemporal dementia (fFTD) and its impact on reproductive decision‐making: A qualitative study
Source: J Genet Couns. 2025 Jan 12;34(1):e2000. doi: 10.1002/jgc4.2000 (PMC11725773; doi:10.1002/jgc4.2000)
Supplement: Supplementary file 1 — Appendix S1 [file JGC4-34-0-s001.docx]

***General information***

*First it would be good to learn a bit more about you.*

*- What age are you?*

*- Do you have any children at the moment?*

*- When they find out about their potential genetic risk of FTD, some people choose to find out what their test results are, and some people choose not to find out. Could I ask which is the case for you?*

***Experiences with FTD and current life circumstances***

*It would be good to start with getting a sense of your experience with your at-risk status and a bit about you.*

- *How do you feel about your at-risk status currently? Has it changed since you first found out?*
- *Could we talk a little bit about your family set-up – who’s in your network?*
- *Who knows about your at-risk status?*

***Experiences with relatives with FTD***

*Sometimes people who are at-risk of frontotemporal dementia will have had some experiences with a relative with frontotemporal dementia. Is that the case for you? (If Yes, continue; if No, proceed to next section). What have your experiences with them been like?*

- *What sort of changes did you notice in your relative?*
- *How did you become aware that they had FTD?*
- *What was your relationship like with your affected relative? Did it change as things progressed?*
- *Are there any specific events that come to mind?*
- *What impact did your experiences with them have on you?*
- *When you think about being at-risk of developing fronto-temporal dementia, are there any particular things about your relative that come to mind?*

***Parenting and Family Planning***

*For participants who have children:*

*You mentioned that you have children. What impacts has being at-risk had on your relationship with your children?*

- *Have you discussed your at-risk status with your partner?*
- *Have you discussed your at-risk status with your children? If so, tell me a bit about how you decided, and how it went?*
- *If you haven’t discussed it with them, do you think you might in the future?*
- *What sort of things do you think are different about being a parent when at-risk of FTD?*
- *Has your relationship with your children changed at all as a result of your at-risk status?*
- *How does your at-risk status influence decisions you make about how you raise your children?*
- *Does it impact how you make plans for the future about/with your children?*
- *Do your experiences with relatives with FTD earlier in life impact how you make decisions about your parenting? How you approach your relationship with your children?*

*For participants who do not have children:*

*You mentioned that you don’t currently have children. Has being at-risk had any impacts on your thoughts about having children in the future?*

- *How does your at-risk status contribute your thinking about having children or not in the future?*
- *Have you discussed your at-risk status with partners? How was it to do this?*
- *Do you think your at-risk status might impact what it is like to be a parent? In what ways?*
- *What sort of things do you think might be different about being a parent when at-risk of FTD?*
- *Do you think your decision about having/not having children might change?*
- *Do your experiences with relatives with FTD earlier in life impact your thinking about having children in the future?*
